# TIA1 oxidation inhibits stress granule assembly and sensitizes cells to stress-induced apoptosis

**DOI:** 10.1038/ncomms10252

**Published:** 2016-01-07

**Authors:** Kyoko Arimoto-Matsuzaki, Haruo Saito, Mutsuhiro Takekawa

**Affiliations:** 1 Division of Molecular Cell Signaling, Institute of Medical Science, The University of Tokyo, Tokyo 108-8639, Japan; 2 Division of Cell Signaling and Molecular Medicine, Institute of Medical Science, The University of Tokyo, Tokyo 108-8639, Japan

## Abstract

Cytoplasmic stress granules (SGs) are multimolecular aggregates of stalled translation pre-initiation complexes that prevent the accumulation of misfolded proteins, and that are formed in response to certain types of stress including ER stress. SG formation contributes to cell survival not only by suppressing translation but also by sequestering some apoptosis regulatory factors. Because cells can be exposed to various stresses simultaneously *in vivo*, the regulation of SG assembly under multiple stress conditions is important but unknown. Here we report that reactive oxygen species (ROS) such as H_2_O_2_ oxidize the SG-nucleating protein TIA1, thereby inhibiting SG assembly. Thus, when cells are confronted with a SG-inducing stress such as ER stress caused by protein misfolding, together with ROS-induced oxidative stress, they cannot form SGs, resulting in the promotion of apoptosis. We demonstrate that the suppression of SG formation by oxidative stress may underlie the neuronal cell death seen in neurodegenerative diseases.

## Abstract (web summary)

Cytoplasmic stress granules (SG) are intracellular aggregates that suppress translation and sequester apoptosis regulatory factors. Here the authors show that reactive oxygen species oxidise the SG-nucleating protein TIA1, preventing SG formation and promoting apoptosis in the presence of additional stress.

## Graphical abstract

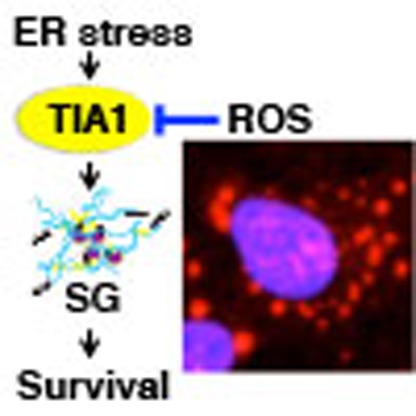

In dealing with environmental stresses, eukaryotic cells either activate defence mechanisms to survive or initiate apoptosis, depending on the level and type of stress. One of the major cellular defence mechanisms is the assembly of cytoplasmic stress granules (SGs). SGs are phase-dense particles that appear when eukaryotic cells are exposed to certain types of stress such as endoplasmic reticulum (ER) stress, heat shock, hypoxia, arsenite or viral infection^1^,^2^,^3^. The core components of SGs are large aggregates of stalled translation pre-initiation complexes that contain mRNA, 40S ribosomal subunits, translation initiation factors and several RNA-binding proteins (RBPs).

Classically, the assembly of SGs is triggered by stress-induced phosphorylation of α-subunit of eukaryotic translation initiation factor 2 (eIF2α), and requires self-aggregation of specific RBPs such as T-cell intracellular antigen-1 (TIA1) or the Ras-GAP SH3 domain-binding protein (G3BP), both of which possess oligomerization domains^4^,^5^. In cells under various stresses, eIF2α is phosphorylated at serine 51 by a number of different stress-sensing kinases such as protein kinase R (PKR), each of which is activated by a different type of stress^1^,^2^. Phosphorylation of eIF2α suppresses productive translation initiation by preventing formation of eIF2-GTP-Met-tRNAi, the ternary complex that joins the initiator Met-tRNAi to the 43S pre-initiation complex. Under the stress conditions, specific RBPs such as TIA1 or G3BP, instead of the ternary complex, interact with an mRNA in the 43S complex, leading to the assembly of a noncanonical, translationally stalled 48S complex. Self-oligomerization of TIA1 or G3BP then promotes the aggregation of these 48S complexes at discrete cytoplasmic foci termed SGs. Therefore, both RNA binding and self-oligomerization of these RBPs are crucial for the assembly of SGs. Indeed, TIA1 possesses three RNA recognition motifs (RRMs) at the NH_2_ terminus, along with a glutamine-rich prion-related domain at the COOH terminus that is responsible for its prion-like self-aggregation^4^.

In addition to the standard mechanism, SGs might be formed by other mechanisms. First, SGs can be nucleated by proteins other than TIA1 and G3BP, such as Caprin-1, the DEAD box family RNA helicase DDX3 and tristetraprolin^6^,^7^,^8^. Most of these proteins possess self-oligomerization domains. Second, some SGs are assembled without eIF2α phosphorylation. Drugs or lipid mediators that target eIF4A, such as pateamine A, hippuristanol and 15d-PGJ2, inhibit translation initiation and initiate SG assembly independently of eIF2α phosphorylation^9^,^10^. These SGs contain the eIF2/5 initiation factors, which are not found in the classical SGs induced by eIF2α phosphorylation^11^. In this article, we will focus on the regulation of the classical SG formation initiated by TIA1.

SGs serve as sites for mRNA storage and triage. While housekeeping mRNAs are recruited to and sequestered into SGs during stress, certain mRNAs encoding proteins involved in stress tolerance (for example, heat shock proteins, molecular chaperones or enzymes involved in damage repair) are excluded from SGs. Therefore, by assembling SGs, cells temporarily reduce the synthesis of housekeeping proteins to prevent accumulation of misfolded proteins, and optimize translation of stress-responsive anti-apoptotic mRNAs. Besides mRNA sorting and translational suppression, SGs sequester several apoptosis regulatory factors into granules and thereby inhibit stress-induced cell death signalling. We have previously reported that when cells are exposed to a SG-inducing stress, the signalling adaptor protein RACK1 is sequestered into SGs, and this sequestration inhibits the stress-activated p38 and JNK pathways and subsequent apoptosis^12^. SGs were shown to recruit regulatory-associated protein of mTOR (Raptor), thereby preventing hyperactivation of mTORC1 signalling and inhibits apoptosis^13^. Recruitment of other signalling molecules (for example, tumour necrosis factor (TNF) receptor-associated factor 2 (TRAF2), rho-associated coiled-coil containing kinase 1 (ROCK1), WD repeat-containing protein 62 (WDR62), protein kinase C α (PKCα), MAP kinase phospho-ser/thr/tyr-binding protein (MK-STYX)) has also been reported^11^,^14^. Thus, formation of SGs serves as a cellular adaptive defence mechanism and protects cells from apoptosis under adverse conditions, by regulating mRNA translation as well as by sequestering signalling molecules. Although many types of stress have been reported to induce SG formation, the assembly of SGs under multiple stress conditions has not yet been elucidated. Furthermore, little is known about the role of SGs in the development of human diseases.

Here we show that oxidative stress inhibits SG formation by oxidizing the SG-nucleating protein TIA1, thereby rendering cells vulnerable to apoptosis. Our data reveal that oxidative stress-mediated suppression of SGs directs cell fate decisions and is involved in the cytotoxicity of polyglutamine (polyQ)-expanded proteins seen in neurodegenerative diseases.

## Results

### H_2_O_2_ inhibits the assembly of SGs

**Figure 1:**
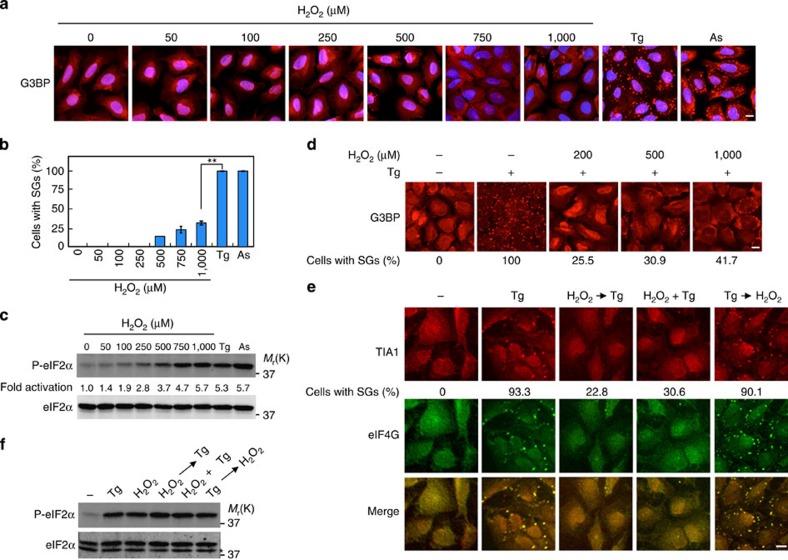
H_2_O_2_ inhibits SG formation. (**a**–**c**) U2OS cells were treated with the indicated concentration of H_2_O_2_, 1 μM thapsigargin (Tg) or 0.5 mM arsenite (As) for 50 min, and SGs were visualized by G3BP immunofluorescence (**a**) and the percentage of cells containing SGs was determined (**b**). Error bars indicate s.e.m. (*n*=3). **P<0.01, Student's *t*-test. In **c**, phosphorylated eIF2α was probed with phospho-eIF2α antibody (upper row). eIF2α protein expression level is shown (lower row). (**d**) U2OS cells were treated with 1 μM Tg together with the indicated concentration of H_2_O_2_ for 50 min. TIA1 and eIF4G were visualized by immunofluorescence and the percentage of cells containing SGs was determined. (**e**,**f**) U2OS cells were treated with 1 μM Tg either alone, 20 min after 200 μM H_2_O_2_ or simultaneously with H_2_O_2_, or were treated with H_2_O_2_ 20 min after Tg. Fifty minutes after Tg treatment SGs were visualized with two SG markers, G3BP (red) and eIF4E (green) (**e**). Phosphorylation level of eIF2α was assessed by immunoblotting using anti-phospho-eIF2α antibody (**f**). *A nonspecific band. Scale bars, 10 μm.

Although many types of stresses including ER stress, arsenite and oxidative stress have been reported to induce SG formation, it remains unclear if there are any differences among these stresses in the efficacy for SG formation. We confirmed that thapsigargin (an ER stress inducer) induced eIF2α phosphorylation that persisted for at least 24 h, and that both thapsigargin and arsenite strongly induced SG assembly in human osteosarcoma U2OS cells (Supplementary Fig. 1a–d). Interestingly, however, treatment of these cells with H_2_O_2_ (oxidative stress) induced little SG formation (Fig. 1a,b), even though H_2_O_2_ induced eIF2α phosphorylation as efficiently as either thapsigargin or arsenite (Fig. 1c). Furthermore, thapsigargin induction of SG formation was significantly impaired in the presence of H_2_O_2_ (Fig. 1d and Supplementary Fig. 1c). H_2_O_2_ suppression of SG formation was seen at a relatively low concentration of H_2_O_2_ (200 μM). H_2_O_2_ treatment also attenuated arsenite induction of SG formation (Supplementary Fig. 1d). Thus, SGs are not formed under oxidative conditions, even if the cells are exposed to SG-inducing stresses.

In theory, H_2_O_2_ might attenuate SG formation by repressing SG assembly or by promoting SG disassembly. To distinguish between these possibilities, we treated cells with H_2_O_2_ and thapsigargin either simultaneously or sequentially in different orders. When H_2_O_2_ was applied 20 min before thapsigargin, or when both reagents were applied at the same time, SG formation was strongly inhibited (Fig. 1e). In contrast, when cells were treated with H_2_O_2_ after SGs are already induced by thapsigargin, these SGs persisted in the presence of H_2_O_2_. Thus, we conclude that H_2_O_2_ impairs the process of SG assembly, but does not promote SG disassembly. H_2_O_2_ treatment did not suppress thapsigargin-induced eIF2α phosphorylation (Fig. 1f). Therefore, H_2_O_2_ inhibits SG assembly without affecting eIF2α phosphorylation, implying that it acts downstream of eIF2α phosphorylation in the process of SG formation.

### TIA1-Cys36 is oxidized by H_2_O_2_

**Figure 2:**
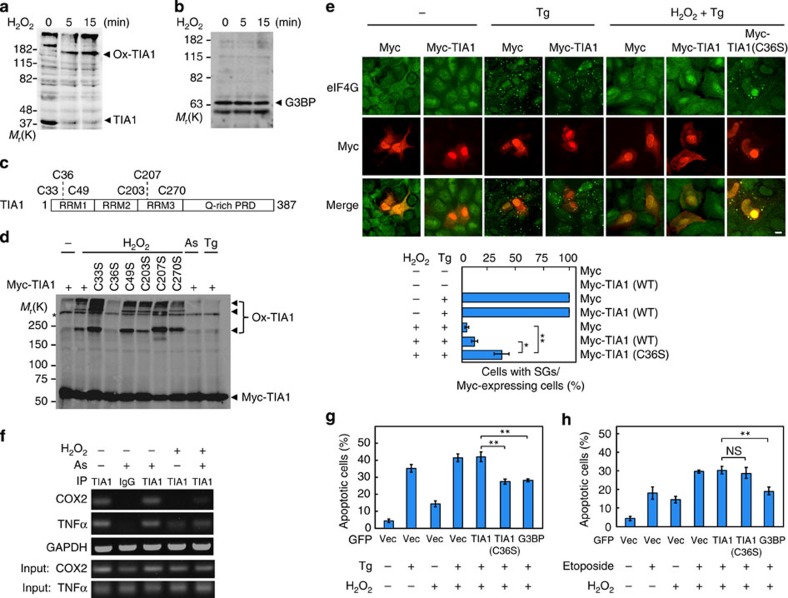
TIA1-Cys36 is oxidized by H_2_O_2_ and SG formation is suppressed. (**a**,**b**) COS cells were stimulated with 1 mM H_2_O_2_ for the indicated times and cell extracts were prepared under non-reducing conditions. (**a**) Endogenous TIA1 was probed by immunoblotting using anti-TIA1 antibody. Ox-TIA1, oxidized TIA1. (**b**) Endogenous G3BP was probed with anti-G3BP antibody. (**c**) Schematic structure of the TIA1 protein. PRD, prion-related domain. (**d**) Myc-tagged TIA1 point mutants were transiently transfected into U2OS cells. After 36 h, the cells were treated with 1 mM H_2_O_2_, 0.5 mM As or 1 μM Tg for 50 min. Cell extracts were prepared and Myc-TIA1 was probed with anti-Myc antibody under non-reducing conditions. (**e**) U2OS cells were transiently transfected as indicated. After 36 h, cells were treated with 1 μM Tg either alone or simultaneously with 200 μM H_2_O_2_ for 50 min. Myc-TIA1 and endogenous eIF4G were visualized by immunofluorescence. Scale bar, 10 μm. The percentage of Myc-expressing cells containing SGs was determined and is shown in the graph. Error bars indicate s.e.m. (*n*=4). *P<0.02, **P<0.01, Student's *t*-test. (**f**) U2OS cells were treated with 200 μM H_2_O_2_ and 0.5 mM as indicated, and were incubated for 50 min. A RIP assay was performed using anti-TIA1 antibody with rabbit IgG as a negative control. (**g**,**h**) Apoptosis induced by Tg was suppressed in cells expressing TIA1(C36S). U2OS cells were transfected as indicated (Vec, vector) and were cultured for 36 h. Cells were then treated with 10 μM Tg and/or 200 μM H_2_O_2_ (**g**) or with 50 μM Etoposide and/or 200 μM H_2_O_2_ (**h**) for another 20 h. To assess apoptosis, cells were stained with Annexin V-Cy3 and visualized by fluorescence microscopy. The number of red-positive apoptotic cells per GFP-positive green cells was determined. About 500 GFP-expressing cells were scored to calculate the percentage of apoptotic cells. Error bars indicate s.e.m. (*n*=4). **P<0.01. NS, not significant, Student's *t*-test.

We next asked how H_2_O_2_ inhibits SG formation. One likely mechanism is the oxidation of the cysteine (Cys) residues of a target protein that is essential for SG formation. Since TIA1 and G3BP are the core proteins for SG assembly^4^,^5^, we initially tested if these proteins are oxidatively modified by H_2_O_2_. For this purpose, COS cells were treated with H_2_O_2_, and cell lysates were separated on SDS–polyacrylamide gel electrophoresis (SDS–PAGE) under non-reducing conditions, followed by immunoblotting to detect TIA1 (Fig. 2a and Supplementary Fig. 2a) and G3BP (Fig. 2b). A marked shift in the electrophoretic mobility of TIA1 (but not of G3BP) to high-molecular-weight species was observed in H_2_O_2_-treated cells. Incubation of the lysates with a reducing agent (2-mercaptoethanol) abolished this mobility shift, indicating that TIA1 is oxidized, resulting in the formation of a covalent complex with (an)other protein(s) through disulfide bonds (Supplementary Fig. 2b). To identify the Cys residue(s) of TIA1 that is/are oxidized, we constructed a series of TIA1 mutants with individual mutations of one of the six Cys residues to serine (Ser) (Fig. 2c). Of these mutants, only TIA1(C36S) showed no band shift following H_2_O_2_ treatment of mutant-transfected cells (Fig. 2d and Supplementary Fig. 2c). Therefore, TIA1-Cys36, which lies in a RRM, is the target of oxidation.

To investigate the relevance of TIA1-Cys36 oxidation to H_2_O_2_-mediated inhibition of SG assembly, we examined the efficiency of SG formation in U2OS cells expressing the oxidation-resistant TIA1(C36S) mutant (Fig. 2e). Treatment with thapsigargin alone efficiently induced SG formation (nearly 100%). When the cells were simultaneously treated with H_2_O_2_ and thapsigargin, almost no SG formation was observed in control cells or in cells that expressed wild-type TIA1 (Myc-TIA1). In contrast, in cells expressing TIA1(C36S), the number of SG-containing cells was significantly increased (up to 40% of the cells). Moreover, TIA1(C36S) also allowed arsenite-induced SG assembly in cells exposed to H_2_O_2_ (Supplementary Fig. 3a). Therefore, H_2_O_2_-mediated inhibition of SG assembly is largely caused by the oxidation of TIA1-Cys36.

The RRM domains of TIA1 bind to AU-rich elements in the mRNA of various genes^15^,^16^. Also, TIA1 homo-oligomerizes through the Q-rich prion-related domain^4^ (Fig. 2c). Both RNA binding and homo-oligomerization of TIA1 are essential for TIA1-mediated SG formation. We therefore determined if either of these TIA1 functions is altered by oxidation. First, we examined the effect of H_2_O_2_ on TIA1 oligomerization in co-immunoprecipitation experiments (Supplementary Fig. 3b). As anticipated, Flag-tagged TIA1 co-precipitated with green fluorescent protein (GFP)-tagged TIA1. This co-precipitation was not reduced by H_2_O_2_, indicating that TIA1 oligomerization is not affected by oxidation. Next, we assessed the effect of TIA1 oxidation on its ability to interact with mRNA, using an RBP immunoprecipitation (RIP) assay. TIA1 was previously shown to bind mRNAs encoding TNFα and cyclooxygenase 2 (COX2)^16^,^17^. These RIP assays showed that the amounts of *TNFα* mRNA and *COX2* mRNA that co-immunoprecipitated with endogenous TIA1 were notably decreased by H_2_O_2_ treatment (Fig. 2f and Supplementary Fig. 3d). In contrast, the oxidation-resistant TIA1(C36S) mutant robustly interacted with these mRNAs even in the presence of H_2_O_2_ (Supplementary Fig. 3c,d). We therefore conclude that oxidized TIA1 at Cys36 loses its ability to bind target mRNAs, resulting in the suppression of SG assembly.

### H_2_O_2_-mediated suppression of SG formation promotes apoptosis

SG formation protects cells during stress by preserving non-translating mRNAs and by sequestering several apoptosis regulatory factors into the granules^2^,^12^. Indeed, augmentation of SG formation in U2OS cells by expression of GFP-G3BP, an efficient inducer of SG formation^5^, suppressed thapsigargin-induced, ER stress-mediated apoptosis, as assessed by Annexin V staining (Supplementary Fig. 4a–c). Conversely, suppression of SG formation by the expression of GFP-G3BP(1-340) or GFP-eIF2α(S51A)^5^,^18^ enhanced thapsigargin-induced apoptosis. We therefore predicted that H_2_O_2_-mediated suppression of SG assembly would promote apoptotic cell death by stresses that would otherwise induce SGs. To test this prediction, GFP-TIA1 or GFP-TIA1(C36S) was transiently expressed in U2OS cells. The cells were then treated with thapsigargin (10 μM) alone or in combination with H_2_O_2_ (200 μM). This concentration of H_2_O_2_ was sufficient to suppress SG formation (Fig. 1d), but was too low to induce apoptosis by itself (Fig. 2g). Annexin V staining showed that combined treatment with thapsigargin and H_2_O_2_ substantially enhanced apoptosis in control (GFP expressing) cells compared with thapsigargin treatment alone (Fig. 2g). Expression of wild-type TIA1 (GFP-TIA1) did not affect the extent of the apoptosis induced by thapsigargin and H_2_O_2_. However, the apoptosis-enhancing effect of H_2_O_2_ was not observed in cells expressing the oxidation-resistant TIA1(C36S) mutant. Furthermore, forced induction of SG formation by the expression of GFP-G3BP^5^ also suppressed thapsigargin and H_2_O_2_-induced apoptosis. In contrast, TIA1(C36S) did not affect apoptosis induced by a combination of etoposide (a SG-non-inducing stress)^12^ and H_2_O_2_ (Fig. 2h). MTT cell viability assay gave similar results (Supplementary Fig. 4d). In summary, inhibition of SG formation by oxidative stress promotes apoptotic cell death by SG-inducing stresses such as ER stress.

### TIA1(C36S) expression suppresses apoptosis in HT22 cells

**Figure 3:**
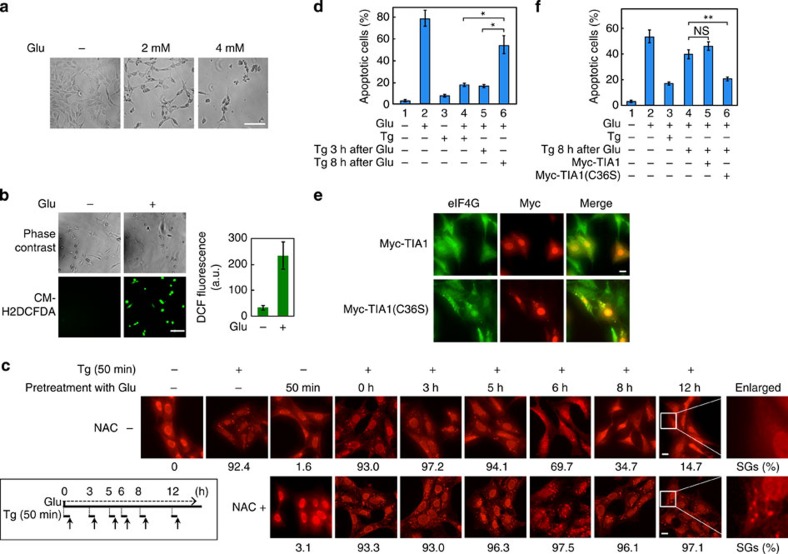
TIA1(C36S) expression restores SG formation under oxidative stress conditions and suppresses apoptosis in HT22 cells. (**a**) HT22 cells were treated with the indicated concentrations of Glu for 24 h. Cell morphology was examined by phase contrast microscopy. (**b**) HT22 cells were incubated with 4 mM Glu for 6 h. ROS production was visualized by fluorescence microscopy using the ROS probe, CM-H_2_DCFDA (lower panels). The 2′,7′-dichlorodihydrofluorescein (DCF) fluorescence intensity (a.u.) was measured for >200 cells and is shown in the graph (right). Error bars indicate s.e.m. (*n*=3). (**c**) HT22 cells were treated with 0.2 μM Tg and 4 mM Glu as indicated. Tg was added either 3, 5, 6, 8 or 12 h after Glu treatment, and SGs were visualized after another 50-min incubation by fluorescence analysis of endogenous G3BP (upper panels). NAC (5 mM) was added to the medium 20 min before Glu treatment (lower panels). (**d**) HT22 cells were treated with 0.2 μM Tg and 4 mM Glu as indicated. Twenty hours after Glu treatment, apoptosis was assayed by staining with Annexin V-Cy3 and visualized by fluorescence microscopy. Error bars indicate s.e.m. (*n*=3). *P<0.02, Student's *t*-test. (**e**) HT22 cells were transfected with either Myc-TIA1 or Myc-TIA1(C36S) and were incubated for 36 h. Cells were then treated with 4 mM Glu and, after 8 h incubation, were treated with 0.2 μM Tg for another 50 min. SGs and Myc-tagged TIA1 were analysed by immunofluorescence microscopy using anti-eIF4G and anti-Myc antibodies, respectively. (**f**) HT22 cells, HT22-Myc-TIA1 or HT22-Myc-TIA1(C36S) stable cell lines were treated with 4 mM Glu and 0.2 μM Tg as indicated. Twenty hours after Glu treatment, apoptosis was measured as in **d**. Error bars indicate s.e.m. (*n*=3). **P<0.01, Student's *t*-test. Scale bars, 100 μm (**a**,**b**) or 10 μm (**c**,**e**).

Both oxidative stress and ER stress have been implicated in the pathogenesis of neurodegenerative disorders, including multiple sclerosis, Alzheimer's disease, Parkinson's disease and so on^19^,^20^,^21^,^22^. A general feature of these disorders is apoptotic neuronal cell death, but its mechanism remains obscure. We therefore tested if oxidative stress contributes to neuronal cell death by inhibiting ER stress-induced SG formation. For this purpose, we initially employed HT22 immortalized mouse hippocampal cell line as a model for the study of glutamate (Glu)-mediated, oxidative stress-induced neuronal cell death. HT22 cells lack functional Glu receptors and are thus not susceptible to Glu-induced excitotoxicity. These cells, however, are still sensitive to high concentrations of extracellular Glu, because Glu induces oxidative stress by inhibiting the Glu/cystine antiporter-mediated uptake of cystine, which is rapidly converted to Cys in the cytoplasm. Lower concentrations of intracellular Cys lead to decreased intracellular glutathione and enhanced accumulation of the ROS^23^,^24^. Exposure of these cells to high concentrations of Glu (2 or 4 mM) induced apoptosis in a concentration-dependent manner (Fig. 3a and Supplementary Fig. 5a). Concomitantly, accumulation of intracellular ROS became detectable 6 h following Glu addition (Fig. 3b). We then examined the effect of Glu-induced oxidative stress on thapsigargin (ER stress)-induced SG formation. Treatment of HT22 cells with thapsigargin (0.2 μM) alone induced strong SG formation within 50 min, whereas Glu administration alone only weakly induced SGs (Fig. 3c). When thapsigargin was added to the culture medium 0, 3 or 5 h after Glu addition, >90% of the cells exhibited robust SG formation. In contrast, when thapsigargin was added as late as 6, 8 or 12 h after Glu administration, when Glu had already induced detectable ROS accumulation (Fig. 3b), the percentage of SG-containing cells was markedly decreased (14.7%; 12 h) (Fig. 3c). This inhibition of SG formation was abrogated by pretreatment of the cells with the antioxidant *N*-acetyl cysteine (NAC), confirming that decreased SG formation was due to Glu-induced ROS production. Thus, in neuronal cells, endogenously generated ROS is sufficient to suppress SG formation.

We next explored the effect of SG formation on Glu-induced, oxidative stress-mediated neuronal cell death. Whereas Glu (4 mM) alone induced apoptosis, a low concentration (0.2 μM) of thapsigargin did not exhibit substantial cytotoxicity for up to 20 h of treatment of HT22 cells (Fig. 3d, lanes 2 and 3). When thapsigargin was added to the cells 0 or 3 h after Glu addition, SGs were formed (Fig. 3c), and Glu-induced apoptosis was markedly suppressed (Fig. 3d, lanes 4 and 5). However, when thapsigargin was added 8 h after Glu, when SG assembly was impaired by ROS, the cells exhibited increased apoptosis (Fig. 3d, lane 6). Similar results were obtained by MTT assay (Supplementary Fig. 5b,c). Thapsigargin did not affect Glu-induced ROS accumulation (Supplementary Fig. 5d). To further confirm that preformed SGs protect neuronal cells from oxidative stress-induced apoptosis, we examined the effect of the oxidation-resistant Myc-TIA1(C36S) on Glu-induced apoptosis. Expression of TIA1(C36S) in HT22 cells restored SG formation (Fig. 3e). TIA1(C36S) also inhibited apoptosis even when thapsigargin was added 8 h after Glu administration (Fig. 3f, lane 6). In contrast, expression of wild-type TIA1 neither restored SG formation (Fig. 3e) nor inhibited apoptosis (Fig. 3f, lane 5). Therefore, when neuronal cells were confronted simultaneously with ER stress and oxidative stress, SG formation was suppressed, and apoptosis was augmented.

### TIA1(C36S) suppresses apoptosis in polyQ70-expressing cells

**Figure 4:**
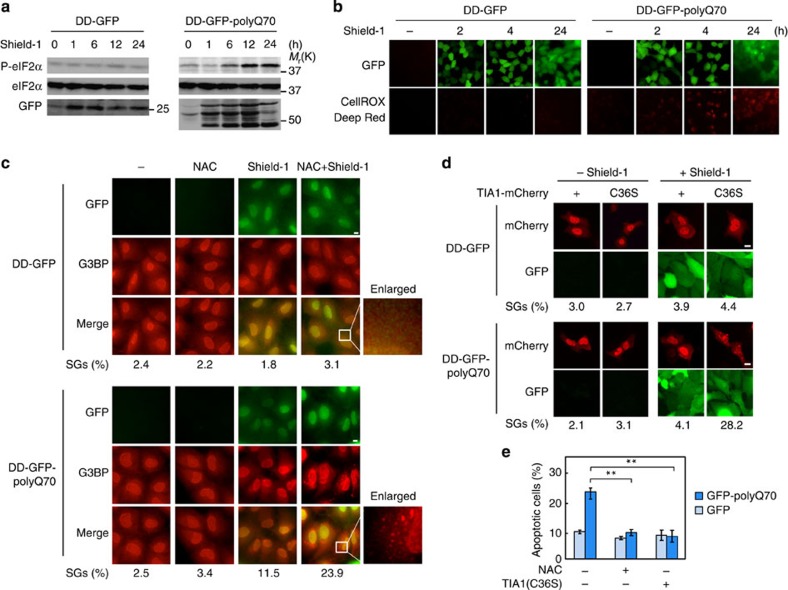
TIA1(C36S) promotes SG formation and suppresses apoptosis in polyQ70-expressing cells. (**a**) U2OS cells stably expressing DD-GFP or DD-GFP-polyQ70 were exposed to 1 μM Shield-1 for the indicated times. Phosphorylated (top) and total (middle) eIF2α in cell lysates were assessed in immunoblotting with specific antibodies. Expression of GFP or GFP-polyQ70 was determined by immunoblotting with an anti-GFP antibody (bottom rows). (**b**) U2OS cells stably expressing DD-GFP or DD-GFP-polyQ70 were exposed to Shield-1 for 2, 4 or 24 h. ROS production was visualized by fluorescence microscopy using the CellROX Deep Red reagent. (**c**) U2OS cells stably expressing DD-GFP (upper panels) or DD-GFP-polyQ70 (lower panels) were treated with NAC, Shield-1 or with both simultaneously as indicated and were incubated for 24 h. GFP expression was detected by fluorescence and endogenous G3BP was visualized by immunofluorescence using anti-G3BP antibody. The percentage of the cells containing SGs was determined and is shown below. (**d**) U2OS cells expressing DD-GFP or DD-GFP-polyQ70 were transfected with mCherry-fusion TIA1 as indicated. Cells were incubated for 24 h and exposed to Shield-1 for 24 h. GFP (green) and mCherry (red) fusion proteins were detected by fluorescence. The percentage of mCherry-expressing cells that contained SGs was determined. (**e**) HEK293 cells transfected with GFP or GFP-polyQ70 were co-transfected with or without TIA1(C36S) as indicated. Twenty four hours after transfection, the medium was replaced with fresh media, with or without NAC, and the cells were incubated for another 4 days. Apoptosis of GFP-positive cells was assessed by Annexin V staining. Error bars indicate s.e.m. (*n*=3). **P<0.01, Student's *t*-test. Scale bars, 10 μm.

Polyglutamine (polyQ) diseases are a group of neurodegenerative disorders that include Huntington's disease and spinocerebellar ataxia^25^,^26^. Their common aetiology is the misfolding and aggregation of disease-associated proteins with an abnormally expanded polyQ stretch, which induces ER stress^27^. PolyQ-expanded proteins also induces cellular ROS production^28^,^29^,^30^. Thus, ER stress and oxidative stress (production of ROS) likely are the main causes of neuronal cell death in polyQ diseases, although other mechanisms have also been proposed, such as cytoskeletal abnormalities, impaired gene expression and dysfunction of axonal transport^31^,^32^,^33^,^34^. To investigate if SG dysfunction is involved in polyQ-induced cell death, we generated a polyQ disease model cell line that inducibly expresses a GFP-tagged ataxin-3-derived fragment containing an expanded polyQ tract (GFP-polyQ70). We fused GFP-polyQ70 to the 12-kDa destabilizing domain (DD) derived from FK506-binding protein^35^. DD-GFP-polyQ70 is normally degraded in the proteasome. However, in the presence of Shield-1, the chemical ligand for DD, the fusion protein is protected from degradation and rapidly accumulates in cells. DD-GFP-polyQ70 was expressed within 1 h following Shield-1 treatment, leading to phosphorylation of eIF2α and ROS production (Fig. 4a,b). In contrast, Shield-1 induced neither eIF2α phosphorylation nor ROS production in the control DD-GFP cells. In this polyQ disease model cell, both oxidative stress and ER stress are induced. We therefore examined if SGs are formed in this model cell. In the absence of Shield-1, SGs were formed in only a small percentage (2.5%) of DD-GFP-polyQ70 cells, whereas following 24 h Shield-1 treatment, the percentage of cells with SGs was moderately increased (11.5%; Fig. 4c). Treatment with both Shield-1 and NAC induced SGs in twice as many cells as were induced by Shield-1 alone (23.9% versus 11.5%). Importantly, the expression of the oxidation-resistant TIA1(C36S), but not of the wild-type TIA1, restored SG formation in DD-GFP-polyQ70-expressing cells (Fig. 4d and Supplementary Fig. 5e,f). We therefore conclude that in this polyQ-disease model cell, ER stress-induced SG formation is countered by concomitant production of ROS that oxidatively inactivates TIA1.

Finally, to examine if suppression of SG formation affects polyQ-induced cytotoxicity, HEK293 cells were transiently transfected with either GFP-polyQ70 or control GFP. Five days after transfection, apoptosis of GFP-positive cells was assessed by Annexin V staining (Fig. 4e). Cells expressing GFP-polyQ70 failed to assemble SGs and underwent substantial apoptosis as compared with control GFP-expressing cells. This polyQ70-induced apoptosis was largely suppressed when SG formation was restored by NAC treatment or by TIA1(C36S) co-expression. Collectively, these findings indicate that suppression of SG formation by ROS contributes to the severe cytotoxicity of polyQ proteins.

## Discussion

In this study, we demonstrated that ROS oxidizes the major SG-nucleating protein TIA1 at Cys36 and consequently suppresses SG assembly by impeding the interaction between TIA1 and its target mRNAs. Previous studies have shown that high concentrations (0.5 mM or more) of H_2_O_2_ induce SG formation^13^,^36^. Our results, however, do not contradict with their findings. According to those studies, high concentrations of H_2_O_2_ induce a breakdown of the eIF4F complex, but little eIF2α phosphorylation. In contrast, arsenite did not induce breakdown of the eIF4F complex. Thus, they proposed that H_2_O_2_ induced SG formation by eIF4F degradation, whereas arsenite induced SG formation by eIF2α phosphorylation. We also found that high concentrations (0.5 mM or more) of H_2_O_2_ induce SG formation, though relatively weakly (Fig. 1a,b). At lower concentrations (for example, 0.2 mM) of H_2_O_2_, SG is not formed, because neither eIF2α phosphorylation (Fig. 1c) nor eIF4F complex breakdown (Supplementary Fig. 6a) occurs. At such low concentrations, H_2_O_2_ suppresses SG formation initiated by other reagents, such as thapsigargin. It is reasonable to assume that H_2_O_2_ should also suppress SG formation by the same mechanism at higher concentrations. However, because of alternative mechanisms of SG formation at high H_2_O_2_ concentrations, the balance will be shifted towards the formation of SGs.

The Cys36 is located within the N-terminal RRMs of TIA1 (Fig. 2c), suggesting that oxidation and the subsequent disulfide bond formation at this site induces conformational alterations of RRMs that are essential for mRNA binding and SG assembly^4^,^18^. Indeed, we demonstrated here that TIA1 oxidation profoundly inhibited its ability to bind mRNAs, explaining why oxidative stress represses SG formation. In this regard, previous studies have shown that a TIA1 mutant that lacks the N-terminal RRMs (TIA1-ΔN) dominantly suppresses SG assembly (Supplementary Fig. 6b)^4^,^18^. Thus, oxidized TIA1 may functionally resemble the dominant inhibitory TIA1-ΔN mutant, in which the N-terminal RRMs are also inactivated by inflame deletion. It should be however noted that because TIA1(C36S) expression did not fully restore SG assembly under oxidative conditions, (an) additional molecule(s) might exist whose oxidation also inhibit(s) SG formation. Indeed some proteins that can be localized in SGs have been reported to be oxidized^37^. Further investigation will be needed to comprehensively understand the molecular mechanisms underlying ROS-induced disturbance of SG formation. While TIA1 controls the nucleation of SGs in the cytoplasm under stress conditions, it also regulates alternative splicing of pre-mRNAs, including those encoding fibroblast growth factor receptor-2 (Fgfr-2) and cystic fibrosis transmembrane conductance regulator (Cftr), in the nucleus under steady-state conditions^38^,^39^,^40^. As both processes require the interaction of TIA1 with mRNAs, oxidative stress not only disrupts SG formation but may also inhibit TIA1-mediated pre-mRNA splicing.

**Figure 5:**
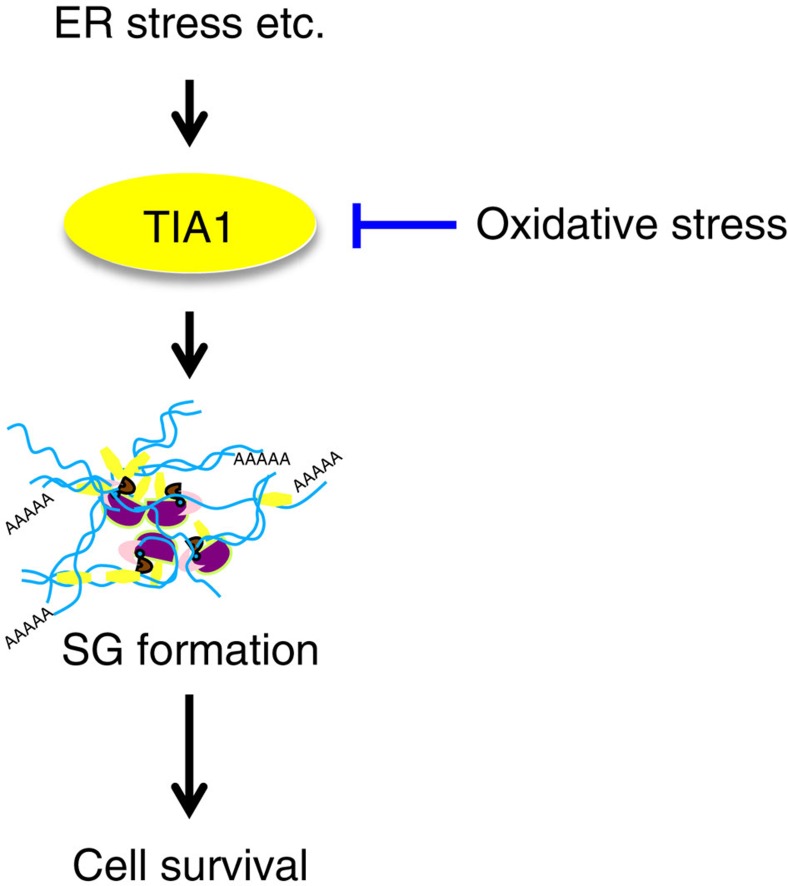
Oxidative stress suppresses SG assembly. When cells are exposed to ER stress, SGs are assembled mainly through homo-oligomerization of TIA1. SG formation is a major adaptive defence mechanism to protect cells from apoptosis. When cells are simultaneously exposed to ER stress and oxidative stress, TIA1 is oxidized and loses its ability to mediate SG formation, resulting in the promotion of apoptosis.

Since the formation of SGs is critical for the regulation of mRNA translation and stress-induced cell death signalling, disturbance of SG assembly would perturb cellular stress responses and dictate cell-fate decisions under stress conditions. In the present study, we found that suppression of SG formation by oxidative stress abolishes its cytoprotective functions and promotes apoptosis caused by SG-inducing stresses (for example, ER stress and arsenite) as well as by SG-non-inducing stresses (for example, etoposide). For example, in neurodegenerative diseases, misfolded proteins evoke both ER stress and oxidative stress. As a result, oxidative stress impairs ER stress-induced SG formation, thereby rendering neuronal cells vulnerable to apoptosis (Fig. 5). Interestingly, several recent studies have reported that although viral infection normally evokes SG formation through virus-induced activation of the eIF2α kinase PKR, the assembly of SGs can be severely compromised by virus-derived RNAs and proteins in cells infected with certain types of pathogenic viruses (for example, West-Nile virus, dengue virus, poliovirus, influenza A virus, human immunodeficiency virus type-1, human T-cell lymphotropic virus type-1, hepatitis C virus and so on)^41^,^42^. West-Nile virus and dengue virus impede SG formation by sequestering TIA1 through specific interaction with the 3′-ends of viral minus-strand RNAs^43^. A poliovirus protein, 3C protease, prevents infection-induced SG assembly by cleaving G3BP (ref. 44). The nonstructural protein 1 of influenza A virus inactivates PKR, thereby preventing SG formation^45^. HIV-1 suppresses SG formation by sequestering a SG-component protein Staufen1 in HIV-1-dependent assembly of ribonucleoprotein complex known as Staufen 1 HIV-1-dependent ribonucleoproteins (SHRNPs)^46^. Since translation of viral proteins and the subsequent viral replication necessarily depend on the host translational machinery, inhibition of SG assembly by pathogenic viruses circumvents SG-mediated translational arrest and promotes viral propagation. Our data demonstrate for the first time that SG assembly can be inhibited not only by extrinsic viral molecules but also by intrinsic oxidative stress, and delineate a novel aetiological aspect of oxidative stress. As oxidative stress is induced by diverse pathological insults, ROS-mediated disturbance of SG assembly may be involved in various pathological processes including neurodegeneration.

In neurodegenerative diseases, recent studies have revealed that several disease-related proteins (TAR DNA-binding protein of 43 kDa (TDP-43), fused in sarcoma (FUS), ataxin-2, survival of motor neuron (SMN) and so on) can associate with SGs, and that SG marker proteins colocalize with pathological inclusion bodies in the patients' brain tissues^47^,^48^,^49^. Although the precise role of the sequestration of SGs into insoluble inclusion bodies remains to be elucidated, it is suggested that pathological inclusions reduce the cytoprotective functions of SGs^50^,^51^,^52^. On the basis of these findings, we propose a hypothetical model that the cytoprotection provided by SGs is compromised in neurodegenerative diseases by two distinct mechanisms, depending on the stage of the disease. In the early stage, oxidative stress represses SG assembly, whereas in the late stage, SGs are inactivated by sequestration into pathological inclusion bodies (Supplementary Fig. 6c). These two mechanisms might synergistically promote neuronal cell death and neurodegeneration. Consistent with our model, a recent report showed that TIA1-positive inclusions can be seen particularly at the mid and late stages in Alzheimer's disease, and increase with disease stage^53^. Since persistent oxidative stress and ER stress play predominant roles in a wide range of human disorders, including diabetes, stroke, atherosclerosis, infection and cancer^54^,^55^,^56^, dysfunction of SGs by oxidative stress might also be involved in the pathogenesis of these human diseases.

## Methods

### Media and buffers

Lysis buffer contains 20 mM Tris-HCl (pH7.5), 1% Triton X-100, 0.5% deoxycholate, 10% glycerol, 137 mM NaCl, 2 mM EDTA, 50 mM β-glycerophosphate, 10 mM NaF, 1 mM sodium vanadate, 1 mM dithiothreitol, 1 mM phenylmethylsulfonyl fluoride (PMSF), 10 μg ml^−1^ leupeptin and 10 μg ml^−1^ aprotinin. SDS–PAGE loading buffer is 65 mM Tris-HCl (pH 6.8), 5% (v/v) 2-mercaptoethanol, 3% SDS, 0.1% bromophenol blue and 10% glycerol. Dithiothreitol and 2-mercaptoethanol were omitted from lysis buffer and SDS–PAGE loading buffer, respectively, when cells were collected under oxidative conditions.

### Plasmids

TIA1, G3BP and eIF2α were subcloned into pcDNA4Myc, pEGFP, pmCherry, pQCXIP (Clontech) and pCMV-Tag2B (Agilent Technologies) by PCR. Mutants were generated by PCR mutagenesis. To create pQCXIP-DD-GFP-polyQ70 construct, polyQ70 was subcloned from pEGFP-ataxin3ΔN-70Q (kindly provided by Dr S. Yanagi, Tokyo University of Pharmacy and Life Science) by PCR.

### Tissue culture and transient transfection

The HT22 hippocampal neuronal cell line was a kind gift from Dr Y. Hirata (Gifu University). Other cell lines used in this study were from our laboratory collection. COS-7, U2OS, HEK293 and HT22 cells were maintained in Dulbecco's modified Eagle's medium supplemented with 10% fetal bovine serum, L-glutamate, penicillin and streptomycin. For transient transfection assays cells were grown in 35-mm dishes and transfected with the appropriate expression plasmids using Lipofectamine LXT (Life Technologies; HT22 cells) or the Effectene transfection reagent (Qiagen; all other cells) according to the manufacturer's protocols.

### Immunofluorescence staining for detecting stress granules

Cells were grown on glass coverslips and transfected with plasmids if necessary. Cellular SG assembly was then stimulated with H_2_O_2_, thapsigargin or sodium arsenite. The cells were then fixed with 1% paraformaldehyde in PBS for 10 min. After washing with PBS, the cells were permeabilized with 0.1% Triton X-100 for 5 min, and incubated in the blocking solution BlockAce (Snow Brand Milk Products) for 1 h. Cells were then incubated with the appropriate antibodies for 50 min in PBS containing 2% BSA. The following antibodies were used: anti-G3BP (sc-81940, Santa Cruz, 1.0 μg ml^−1^); anti-TIA1 (sc-166247, Santa Cruz, 4.0 μg ml^−1^); anti-eIF4G (A300-502A, Bethyl Lab, 10.0 μg ml^−1^); anti-eIF4E (sc-9976 and sc-13963, Santa Cruz, 10.0 μg ml^−1^); and anti-Myc (sc-40, Santa Cruz, 2.0 μg ml^−1^). The cells were washed three times with PBS and incubated with an Alexa Fluor 546-conjugated rabbit anti-mouse antibody and/or a Cy2-conjugated goat anti-rabbit antibody for 30 min. The stained cells on coverslips were washed three times with PBS and were mounted in FluorSave Reagent (Calbiochem). Cells were stained with DAPI (25 ng ml^−1^ in PBS) to visualize the nuclei. SG formation was assessed by determination of the number of cells expressing at least two SGs per cell.

### Detection of apoptotic cells by Annexin V-Cy3

Early and late apoptotic cells were detected using an Annexin V-Cy3 apoptosis detection kit (Biovision) according to the manufacturer's instructions. Briefly, 2 × 10^5^ cells were treated where indicated with thapsigargin, H_2_O_2_, etoposide or glutamate and were incubated for 20 h. Cells were then collected and resuspended in the binding buffer, followed by incubation with Annexin V-Cy3 in the dark for 5 min. Apoptotic cells were identified and scored by direct visualization of red-coloured membrane staining under a fluorescence microscope. Four to six representative fields of at least 100 cells were scored.

### Fluorescence microscopy

Fluorescence microscopic images of fixed cells were captured using a Nikon TE2000-E fluorescent microscope equipped with a Photometrics Cool SNAP HQ CCD camera, or an inverted Olympus IX81 microscope equipped with a OImaging Retiga EXi digital camera (IEEE1394) and the Universal Imaging Metamorph software (Molecular Devices).

### RIP assay

The RIP assay using rabbit polyclonal anti-TIA1 antibody (RN014P, MBL) was performed using the Magna RIP RNA-Binding Protein Immunoprecipitation Kit (Millipore). The cell pellet from 2 × 10^7^ U2OS cells was suspended in 100 μl of RIP lysis buffer. The cell lysates were incubated with 5 μg of either anti-TIA1 antibody or rabbit IgG attached to magnetic beads in RIP immunoprecipitation buffer overnight at 4 °C. After washing six times, the beads were incubated with proteinase K for 30 min at 55 °C. After centrifugation, RNA was purified from the supernatant by extraction with phenol:chloroform:isoamyl alcohol followed by ethanol precipitation. RT–PCR was performed by using the SuperScript III One-Step RT–PCR System with Platinum Taq (Invitrogen) and the following primer sets.

COX2 forward and reverse oligonucleotides:

5′-gaatcattcaccaggcaaattg-3′ and 5′-tttctgtactgcgggtggaac-3′

TNFα forward and reverse oligonucleotides:

5′-cccagggacctctctctaatc-3′ and 5′-atgggctacaggcttgtcact-3′

GAPDH forward and reverse oligonucleotides:

5′-accacagtccatgccatcac-3′ and 5′-ccaccaccctgttgctgta-3′.

PCR products were visualized by agarose gel electrophoresis.

Quantitative RT–PCR analysis was performed with SYBR green (Roche) and the ABI7500 real-time PCR system (Applied Biosystems). The following primer sets were used.

COX2 forward and reverse oligonucleotides:

5′-cccttgggtgtcaaaggtaa-3′ and 5′-gccctcgcttatgatctgtc-3′

GAPDH forward and reverse oligonucleotides:

5′-ccccttcattgacctcaactac-3′ and 5′-gatgacaagcttcccgttctc-3′

For U2OS cells stably expressing Myc-TIA1(C36S), RIP assay was performed by using mouse monoclonal anti-Myc-Tag antibody (MBL, clone PL14) in the same way as above.

### ROS measurement

ROS levels in HT22 and U2OS were determined utilizing either CM-H2DCFDA or the CellROX Deep Red Reagent (Life Technologies), respectively, according to the manufacturer's protocols. HT22 cells were cultured in a glass-bottomed dish with phenol red-free DMEM. Cells were incubated with 4 mM Glu for 6 h. Culture medium was aspirated and the cells were then treated with 4 μM CM-H2DCFDA in PBS for 40 min. The PBS containing the probe was replaced with phenol red-free DMEM, and the cells were incubated for another 1 h. Green fluorescence was observed by fluorescence microscopy. U2OS-DD-GFP and U2OS-DD-GFP-polyQ70 cells were cultured in glass bottom dishes and were treated with Shield-1. After incubation for the indicated times, 5 μM CellROX Deep Red reagent was added and incubated for another 30 min. Cells were then washed three times with PBS and the red fluorescence emitted by CellROX was visualized by microscopy. NAC (Sigma) was used to inhibit ROS.

### Generation of DD-GFP-polyQ70 stable cell lines

The retroviral expression vector, pQCXIP-DD-GFP, was generated to express a fusion protein of GFP and the DD. DD was subcloned from the pPTuner Vector (TaKaRa). For generation of pQCXIP-DD-GFP-polyQ70, the cDNA fragment encoding polyQ was inserted into pQCXIP-DD-GFP. GP2-293 cells were transfected with pQCXIP-DD-GFP or pQCXIP-DD-GFP-polyQ70 each together with pVSV, a packaging construct, using Effectene. Viral supernatants were collected after 48 h and U2OS cells were infected with the concentrated supernatants supplemented with 10 μl ml^−1^ polybrene for 4 h at 37 °C, followed by selection with 3 μg ml^−1^ puromycin.

### Cell proliferation assay

Cell viability was evaluated using MTT assays. Cells were seeded in triplicate on 96-well plates at a density of 5.0 × 10^3^ cells per well, followed by incubation at 37 °C for 24 h with appropriate combinations of stimuli. Cell Count Reagent SF (10 μl; Nacalai Tesque) was then added into each well and the cells were incubated for 1 h at 37 °C. The optical density of the culture solution in the plate was measured using a microplate reader (Molecular Devices) at 450 nm.

### Co-immunoprecipitation assay of protein binding

Cell lysates were prepared in lysis buffer and were incubated with protein G-Sepharose beads at 4 °C for ∼12 h. Precleared lysates were incubated with an appropriate antibody at 4 °C for 4 h with gentle rotation. BSA-blocked protein-G-Sepharose beads were then added and incubation was continued for another 1 h. Immunoprecipitates were collected by centrifugation, washed five times with lysis buffer and subjected to SDS–PAGE.

### 7-methyl GTP (m7GTP) pull-down assay

U2OS cells were treated with 200 μM or 1 mM H_2_O_2_ for 50 min, and whole-cell lysate was collected with lysis buffer (50 mM Tris-HCl (pH 7.2), 100 mM NaCl, 1 mM EDTA and 0.5% NP-40) supplemented with protease inhibitors. For eIF4E pull-down assay, 20 μl m7GTP agarose beads (γ-Aminophenyl-m^7^GTP (C_10_-spacer)-Agarose, Jena Bioscience) were washed twice with RNase-free buffer (15 mM Tris-HCl (pH 7.0), 100 mM NaCl and 1 mM EDTA). Cell lysates were then incubated with beads (20 μl of beads for each sample) on the rotator overnight at 4 °C. After incubation, beads were washed with lysis buffer three times. Proteins bound to m7GTP beads were eluted with SDS–PAGE loading buffer and analysed by western blotting.

### Immunoblotting analyses

Appropriate amounts of proteins were resolved by SDS–PAGE and transferred onto nitrocellulose membranes. After blocking with 4.0% skim milk, membranes were probed with appropriate antibodies and visualized using enhanced chemiluminescence detection. The following antibodies were used: anti-Flag mAb M2 (F1804, Sigma); anti-Myc mAb (sc-40, Santa Cruz); anti-4E-BP1 rabbit mAb (9644, Cell Signaling); polyclonal anti-Myc (sc-789, Santa Cruz); polyclonal anti-phospho-eIF2α (9721, Cell Signaling), polyclonal anti-eIF2α (9722, Cell Signaling); anti-TIA1 (RN014P, MBL); polyclonal anti-G3BP (sc-81940, Santa Cruz); polyclonal GFP (#598, MBL); polyclonal anti-RFP (PM005, MBL); polyclonal anti-eIF4G (A300-502A, Bethyl Lab); and polyclonal anti-eIF4E (sc-13963, Santa Cruz). All these antibodies were used at 1:1,000 dilution. Full scans of representative immunoblots are presented in Supplementary Figs 7 and 8.

### Statistics

The statistical significance of the difference between mean values was tested using Student's *t*-test. Data are means±s.e.m. of at least three independent experiments.

## Additional information

**How to cite this article:** Arimoto-Matsuzaki, K. *et al.* TIA1 oxidation inhibits stress granule assembly and sensitizes cells to stress-induced apoptosis. *Nat. Commun.* 7:10252 doi: 10.1038/ncomms10252 (2016).

## Supplementary Material

Supplementary Information Supplementary Figures 1-8
