# Supplementary material for: TIA1 oxidation inhibits stress granule assembly and sensitizes cells to stress-induced apoptosis
Source: Nat Commun. 2016 Jan 7;7:10252. doi: 10.1038/ncomms10252 (PMC4729832; doi:10.1038/ncomms10252)
Supplement: Supplementary Information — Supplementary Figures 1-8 [file ncomms10252-s1.pdf]

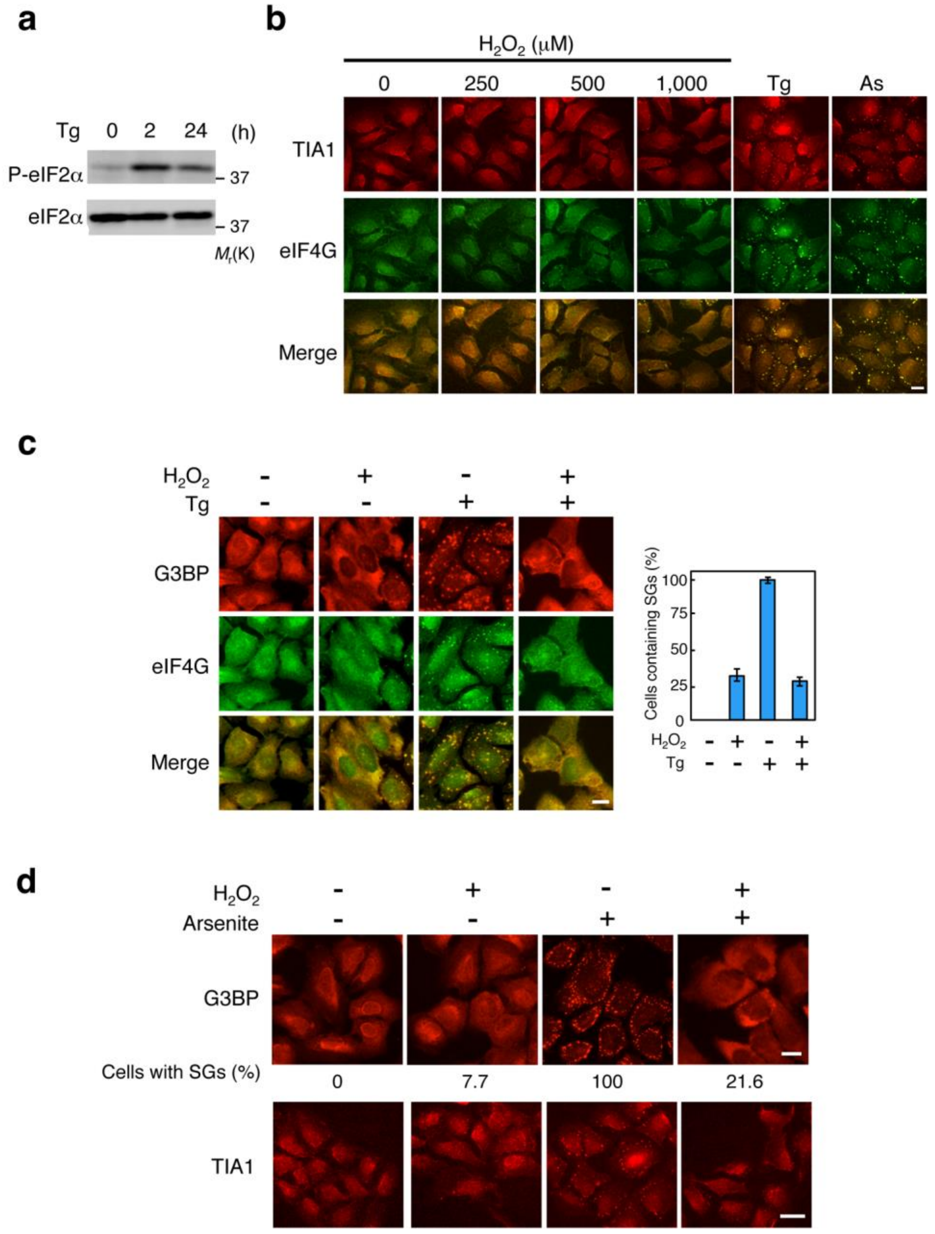

Supplementary Figure 1. H_2O_2 suppresses SG formation.
(**a**) U2OS cells were treated with 1 μM thapsigargin (Tg) for the indicated times. Phosphorylated eIF2α was probed with phospho-eIF2α antibody (upper row). The expression level of total eIF2α is shown in the lower row.
(**b**) U2OS cells were treated with the indicated concentration of H_2O_2, 1 μM Tg, or 0.5 mM arsenite (As) for 50 min, and SGs were visualized by TIA1 and eIF4G immunofluorescence.
(**c**) U2OS cells were treated with 1 mM H_2O_2 or 1 μM Tg as indicated for 50 min. Stress granule markers, G3BP (red) and eIF4G (green), were visualized by immunofluorescence. The percentage of cells containing SGs is shown at right. Error bars indicate s.e.m. (n=4).
(**d**) U2OS cells were treated with 200 μM H_2O_2 and/or 0.5 mM arsenite as indicated, and, after 50 min incubation, G3BP or TIA1 was visualized and SG-containing cells were counted. The scale bars in (**b**), (**c**) and (**d**) represent 10 μm.

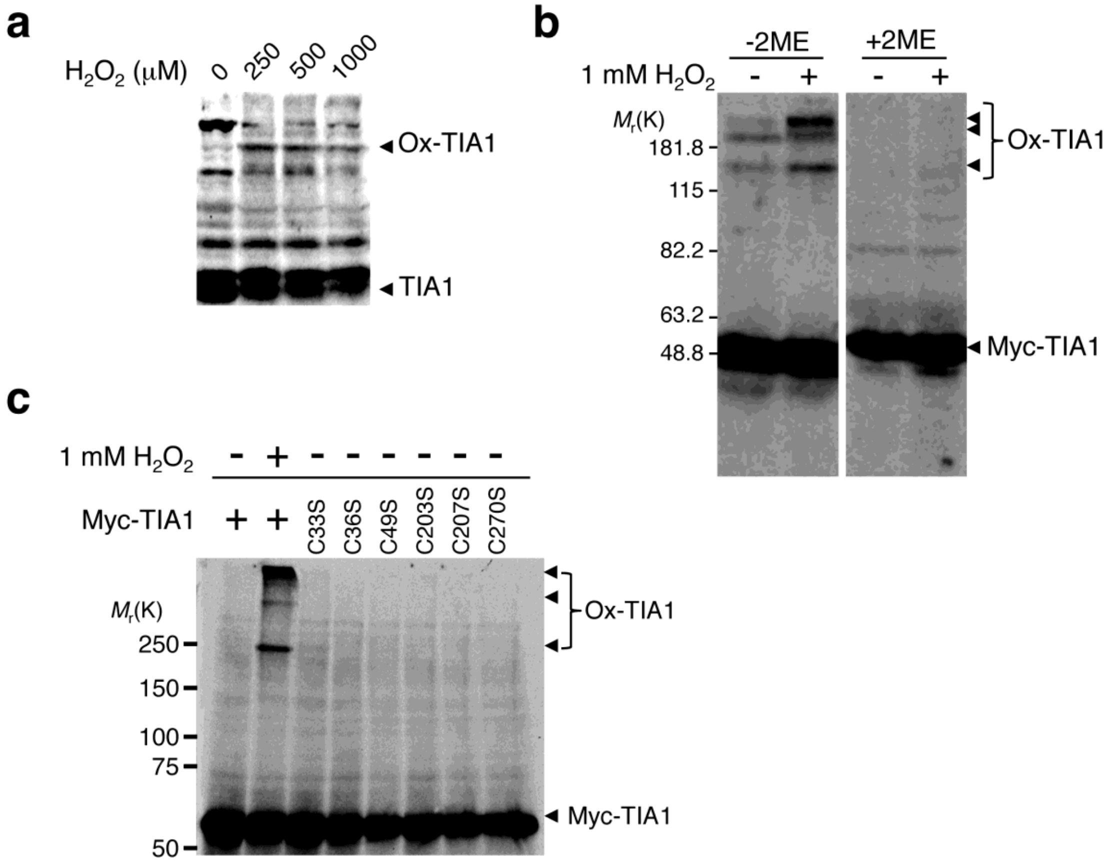

Supplementary Figure 2. TIA1 is oxidized by H_2O_2.

(**a**) COS cells were stimulated with the indicated concentrations of H_2O_2 for 15 min and cell extracts were prepared under non-reducing conditions. Endogenous TIA1 was probed by immunoblotting using anti-TIA1 antibody. Ox-TIA1, oxidized TIA1.

(**b**) Myc-tagged TIA1 was transiently transfected into U2OS cells. After 36 h, cells were treated with 1 mM H_2O_2 for 50 min. Cell extracts were separated under non-reducing (-2ME) or reducing (+2ME) conditions and Myc-TIA1 was immunoblotted with anti-Myc antibody. Ox-TIA1: oxidized TIA1.

(**c**) Myc-TIA1, either wild-type or the indicated mutants, were transiently transfected into U2OS cells. Thirty-six hours after transfection, cells were treated (+) or not treated (-) with 1 mM H_2O_2 for 50 min. Cell extracts were prepared and Myc-TIA1 was probed with anti-Myc antibody under non-reducing conditions.

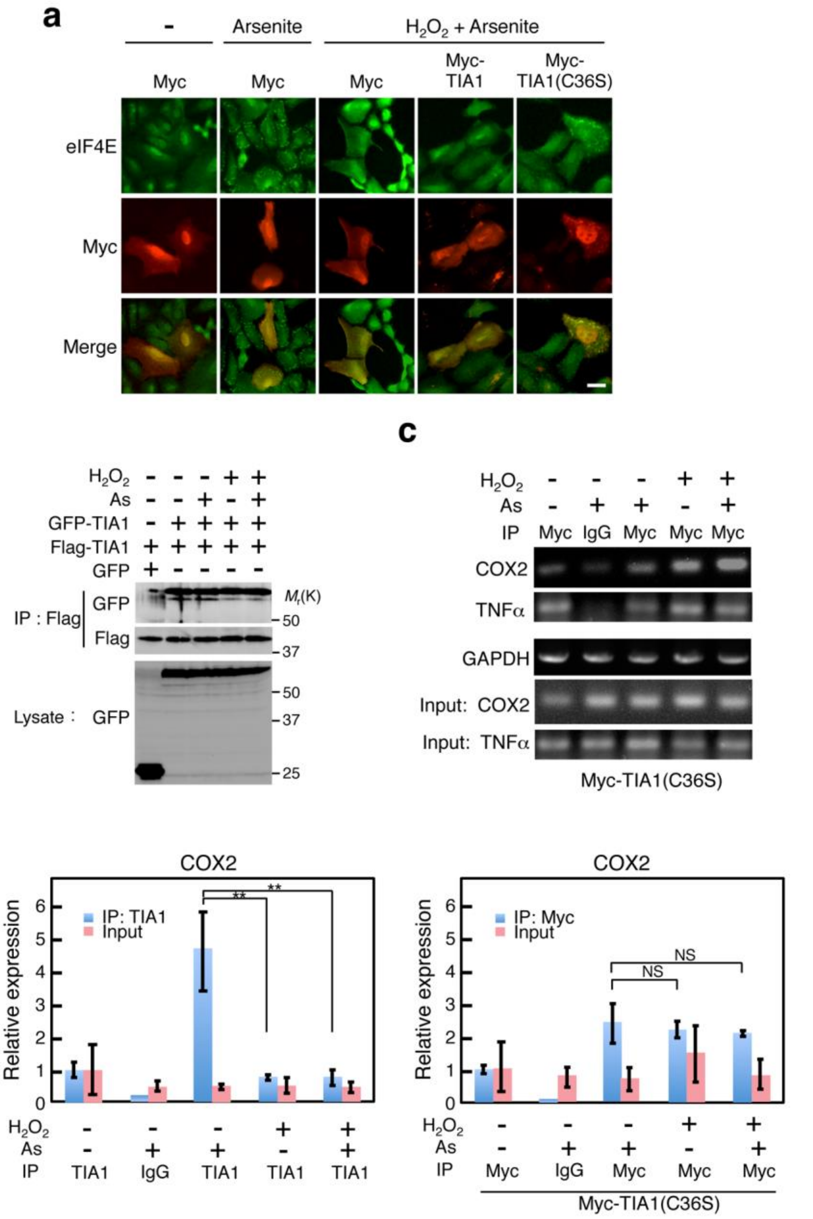

Supplementary Figure 3. TIA1(C36S) expression promotes SG formation.

(**a**) Cells expressing TIA1(C36S) form SGs. U2OS cells were transiently transfected as indicated. After 36 h, cells were treated with 0.5 mM arsenite (As) alone or simultaneously with 200 µM H_2O_2 for 50 min. Endogenous eIF4E (green) and Myc-TIA1 (red) were visualized by immunofluorescence. The scale bar represents 10 µm.

(**b**) H_2O_2 does not affect TIA1 oligomerization. Cytoplasmic extracts were prepared under non-reducing conditions from COS-7 cells transfected with GFP-TIA1, Flag-TIA1 or GFP as indicated. Extracts were immunoprecipitated using a Flag antibody and co-immunoprecipitated GFP-TIA1 was detected by immunoblotting (top row).

(**c**) U2OS cells stably expressing Myc-TIA1(C36S) were treated with 200 µM H_2O_2 and 0.5 mM As as indicated, and were incubated for 50 min. RIP assay was performed using an anti-Myc antibody with mouse IgG as a negative control.

(**d**) U2OS cells or U2OS cells stably expressing Myc-TIA1(C36S) were treated with 200 µM H_2O_2 and 0.5 mM As as indicated, and were incubated for 50 min. A RIP assay was performed using anti-TIA1 or anti-Myc antibody and quantitative RT-PCR was performed to quantify the level of co-precipitated *COX2* mRNA. Error bars indicate s.e.m. (n=3). $^{**}P < 0.05$, Student's *t*-test.

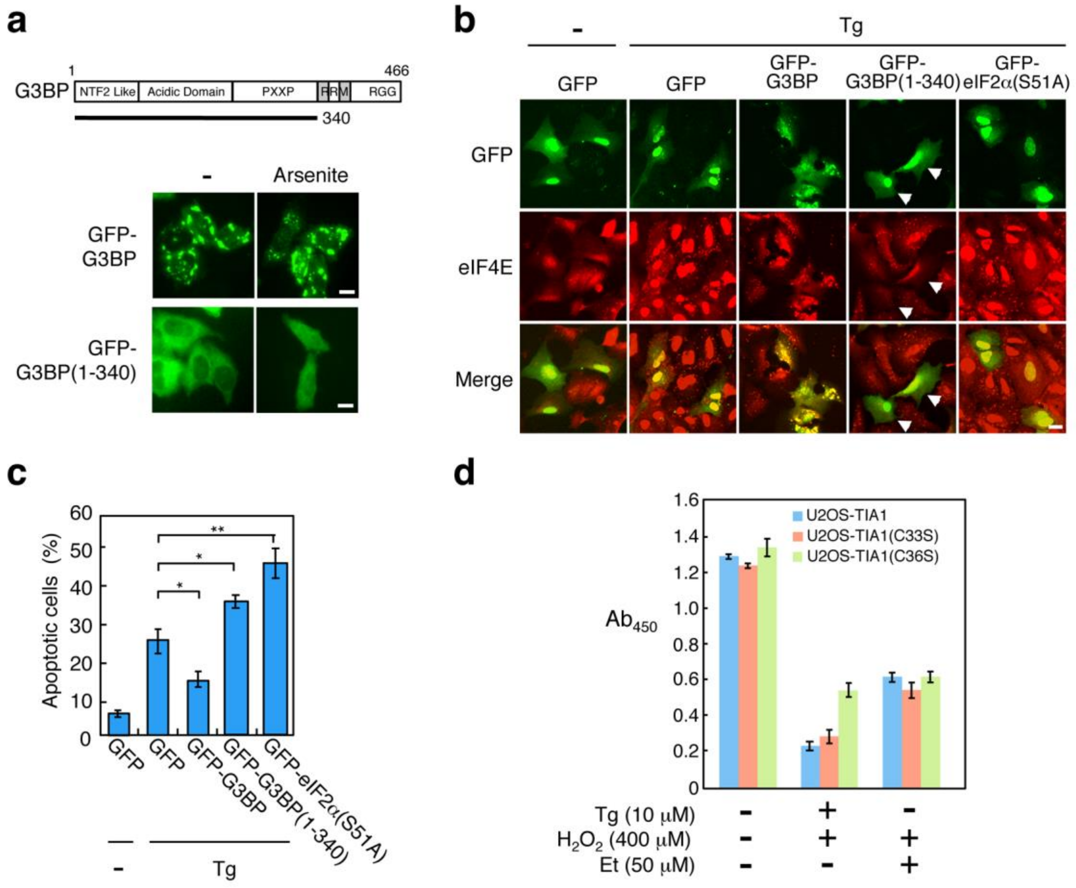

Supplementary Figure 4. TIA1(C36S) expression suppresses apoptosis.

(**a**) Schematic structure of the G3BP protein. RRM, RNA recognition motif; RGG, arginine-glycine rich motif (upper figure). Overexpression of GFP-G3BP alone is sufficient to induce SG formation, while expression of G3BP(1-340) suppresses SG formation. U2OS cells were transfected with GFP-G3BP (upper panel) or GFP-G3BP(1-340) (lower panel) and incubated for 36 h. GFP fluorescence was detected by microscopy. The scale bars represent 10 μm.

(**b-c**) Inhibition of SG formation promotes apoptosis. (**b**) U2OS cells were transfected as indicated were incubated for 36 h. Cells were then treated with 10 μM Tg for 50 min, and transfected proteins and SGs were visualized by GFP (green) and immunofluorescence using anti-eIF4E antibody (red) respectively. The scale bar represents 10 μm. (**c**) To assess apoptosis, transfected cells were incubated for 20 h with 10 μM Tg, and apoptotic cells were then stained with Annexin V-Cy3 and visualized by fluorescence microscopy. The number of red-positive apoptotic cells per GFP-positive green cells was determined. The percentage of apoptotic cells is shown. Error bars indicate s.e.m. (n=4). $*P < 0.05$, $**P < 0.005$, Student's *t*-test.

(**d**) Apoptosis induced by Tg was suppressed in cells expressing TIA1(C36S). U2OS cells stably expressing TIA1, TIA1(C33S), or TIA1(C36S) were treated with Tg, H_2O_2 or etoposide (Et) as indicated. After 20 h incubation, cell viability was assayed by MTT assay. Ab_{450}: absorbance at 450 nm. Error bars indicate s.e.m. (n=3).

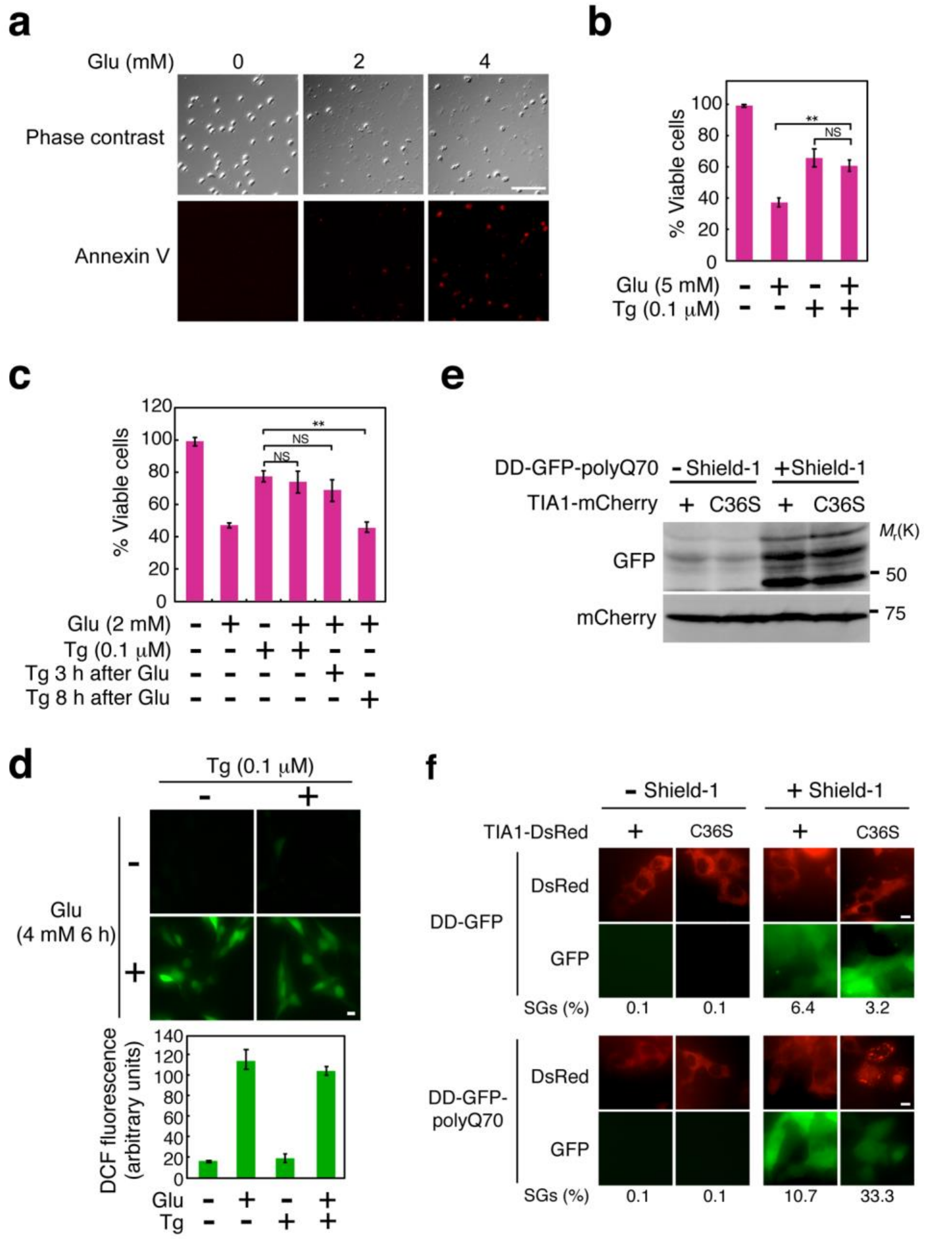

Supplementary Figure 5. SG formation protects neuronal cells from oxidative stress-induced apoptosis.

(**a**) HT22 cells were treated with the indicated concentrations of Glu for 24 h. Apoptotic cells were visualized by Annexin V-Cy3 staining. The scale bar represents 100 μm.

(**b** and **c**) SG formation suppresses apoptosis. HT22 cells were treated with glutamate (Glu) or Tg as indicated. Twenty hours after Glu addition, MTT assay was performed and the percentage of viable cells was calculated. Error bars indicate s.e.m. (n=4). $^{**}P < 0.01$. NS, not significant, Student's *t*-test.

(**d**) Tg does not inhibit ROS production. HT22 cells were treated with 0.1 μM Tg or 4 mM Glu as indicated and after 6 h, ROS production was visualized by fluorescence microscopy using ROS probe, CM-H_2DCFDA (left). The DCF fluorescence intensity (arbitrary units) was measured for >200 cells and is shown in the graph (right). Error bars indicate s.e.m. (n=3). The scale bar represents 10 μm.

(**e**) U2OS cells stably expressing DD-GFP-polyQ70 were transfected with mCherry-fusion TIA1 (wild-type or a C36S mutant) as indicated. Cells were incubated for 24 h and exposed to Shield-1 for another 24 h. Cell extracts were then prepared and GFP-polyQ70 and TIA1-mCherry were probed with anti-GFP and anti-RFP antibodies, respectively.

(**f**) U2OS cells expressing DD-GFP or DD-GFP-polyQ70 were transfected with DsRed-fusion TIA1 as indicated. Cells were incubated for 24 h and exposed to Shield-1 for 24 h. GFP (green) and DsRed (red) fusion proteins were detected by fluorescence. The percentage of DsRed-expressing cells that contained SGs was determined.

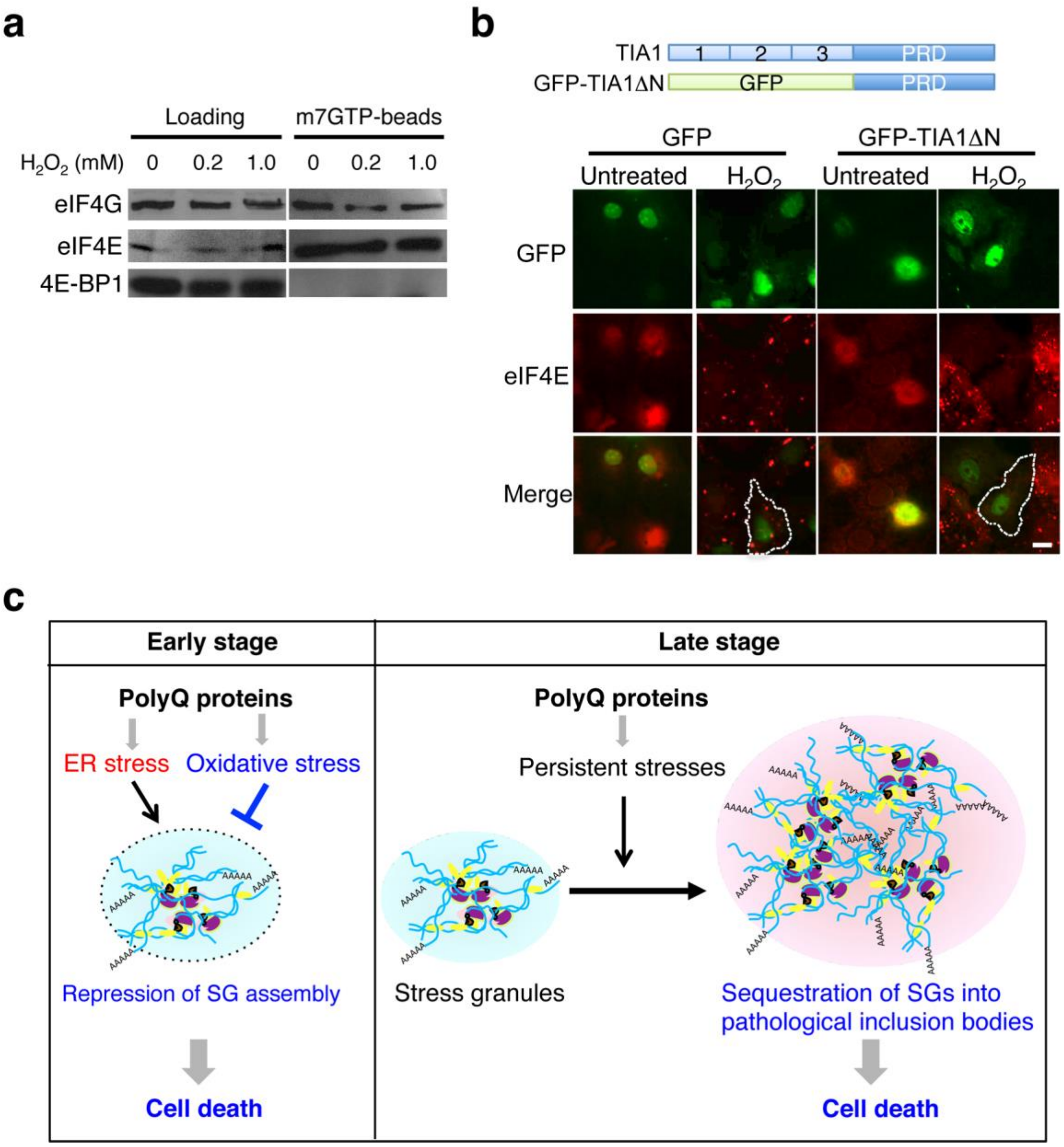

Supplementary Figure 6. Oxidative stress suppresses SG assembly and promote apoptotic cell death.

(**a**) U2OS cells were treated with the indicated concentrations of H_2O_2 for 50 min. The eIF4F complexes were pulled-down from cell lysate using 7-methyl GTP (m7GTP)-beads. The amounts of eIF4G, eIF4E, and 4E-BP1 in the eIF4F complex were determined by immunoblotting using respective antibodies.

(**b**) A dominant-negative TIA1ΔN mutant inhibits SG assembly. COS-7 cells were transiently transfected with either GFP alone or a GFP-TIA1-ΔN, which is an N-terminally truncated TIA1 mutant that lacks RRMs. After 48 h, the cells were treated with a high concentration of H_2O_2 (1 mM) for 50 min. Endogenous eIF4E, a SG marker, was visualized by immunofluorescence.

(**c**) A hypothetical model of neurodegenerative diseases. In neurodegenerative diseases, the cytoprotection provided by SGs is compromised by two distinct mechanisms. In the early stage, oxidative stress represses SG assembly, whereas in the late stage, SGs are inactivated by sequestration into pathological inclusion bodies.

Fig. 1c

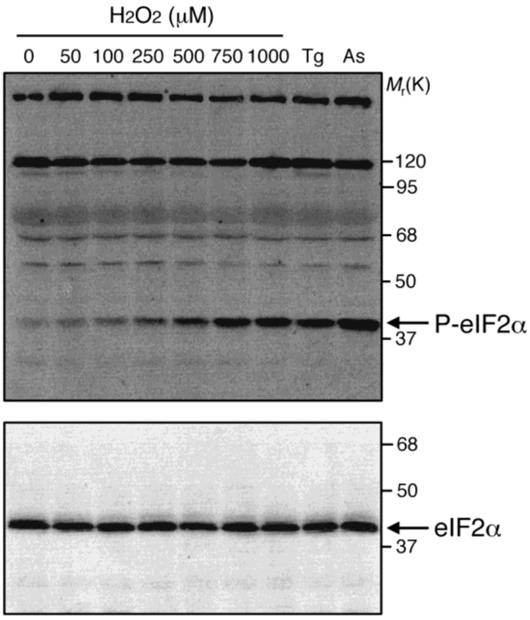

Fig. 1f

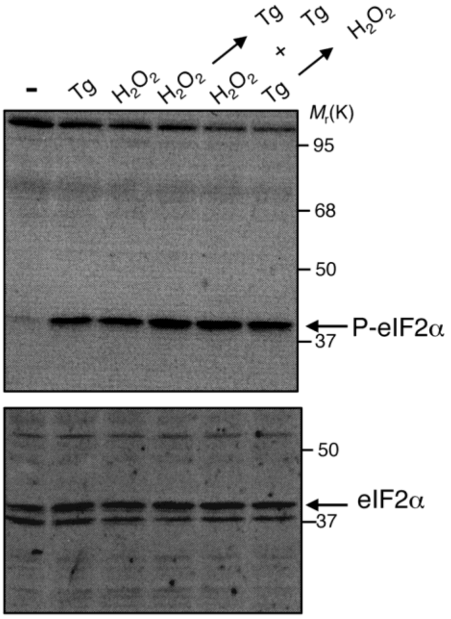

Supplementary Figure 7.

Uncropped scans of the immunoblots in Figures 1c and 1f.

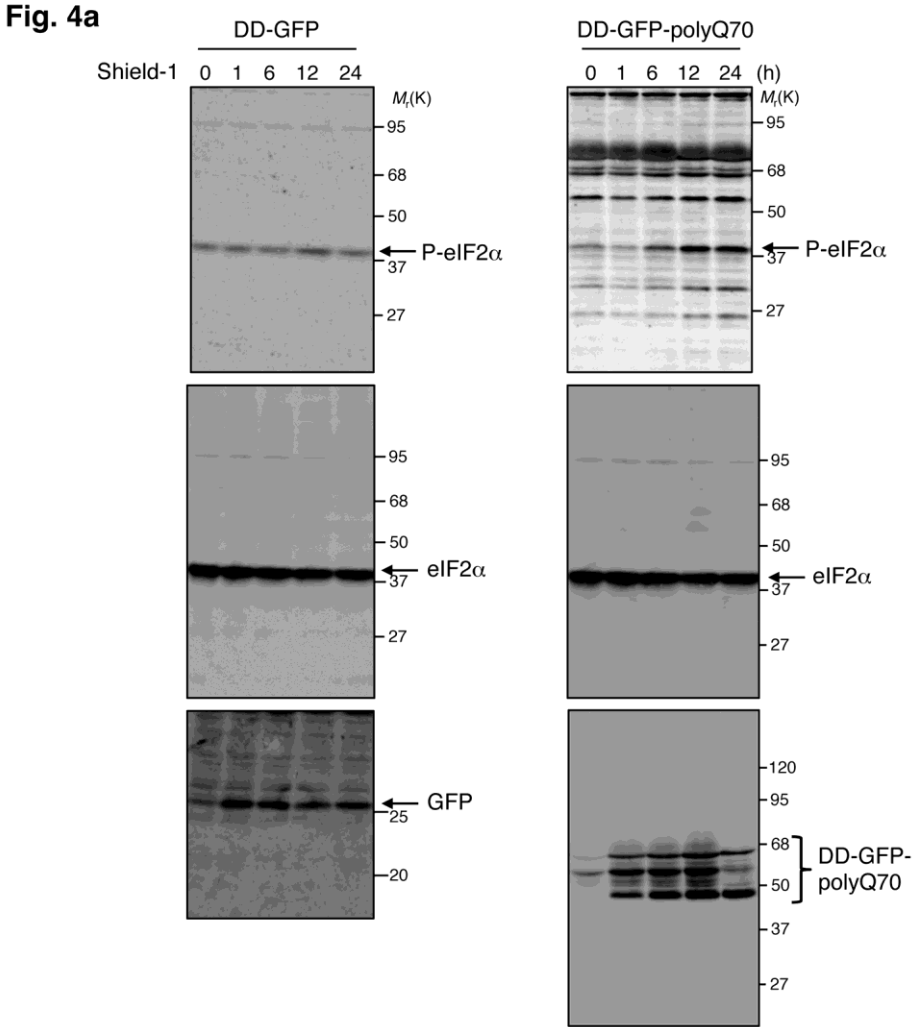

Supplementary Figure 8.
Uncropped scans of the immunoblots in Figure 4a.
